# Unveiling the clinical signs and pathology in red deer (*Cervus elaphus*) naturally infected with epizootic haemorrhagic disease virus serotype 8

**DOI:** 10.1186/s13567-026-01807-w

**Published:** 2026-07-01

**Authors:** Leonor Muñoz-Fernández, Irene Agulló-Ros, David Cano-Terriza, Fatma Abo-Zakaib-Ali, Remigio Martínez, Mario Frías, Jose Antonio Ortiz, Christian Gortázar, Gisella Armillotta, Maria Teresa Mercante, Elena Fuentes-Rodríguez, Jose Francisco Ruiz-Fons, Ignacio García-Bocanegra, Roser Velarde, Mª Ángeles Risalde

**Affiliations:** 1 Departamento de Anatomía y Anatomía Patológica Comparadas y Toxicología, Grupo de Investigación en Sanidad Animal y Zoonosis (GISAZ), UIC Zoonosis y Enfermedades Emergentes (ENZOEM), Centro Andaluz de Investigación de Zoonosis y Enfermedades Emergentes (CAIZEM), Edificio Ramón y Cajal,, Universidad de Córdoba, Campus de Rabanales, 14014 Córdoba, Spain; 2 Departamento de Sanidad Animal, Grupo de Investigación en Sanidad Animal y Zoonosis (GISAZ), UIC Zoonosis y Enfermedades Emergentes (ENZOEM), Universidad de Córdoba, 14014 Córdoba, Spain; 3 CIBERINFEC, ISCIII – CIBER de Enfermedades Infecciosas, Instituto de Salud Carlos III, 28029 Madrid, Spain; 4 Department of Pathology and Clinical Pathology, Faculty of Veterinary Medicine, Sohag University, Sohag, 82524 Egypt; 5 Medianilla S.L., Finca Las Lomas, Las Lomas, Vejer de La Frontera, 11179 Cádiz, Spain; 6 Grupo Sanidad y Biotecnología (SaBio), Instituto de Investigación en Recursos Cinegéticos (IREC), CSIC-UCLM-JCCM, 13005 Ciudad Real, Spain; 7 Istituto Zooprofilattico Sperimentale Dell’Abruzzo E del Molise Giuseppe Caporale (IZS), 64100 Teramo, Italy; 8 Departamento de Investigación, Fundación Artemisan, 13001 Ciudad Real, Spain; 9 Servei d’Ecopatologia de Fauna Salvatge (SEFaS), Departament de Medicina I Cirurgia Animals, Universitat Autònoma de Barcelona (UAB), Bellaterra, 08193 Barcelona, Spain

**Keywords:** *Cervus elaphus*, lesions, EHDV-8, pathogenesis, symptoms, morbidity, mortality

## Abstract

Epizootic haemorrhagic disease (EHD) is a notifiable disease to the World Organisation for Animal Health caused by *Orbivirus ruminantium* (EHDV), closely related to bluetongue virus (BTV) and transmitted by *Culicoides* spp. In autumn 2022, EHDV serotype 8 (EHDV-8) was detected for the first time in Spain and Europe, affecting domestic and wild ruminants. Red deer (*Cervus elaphus*) were the most affected wild species, with reports of neurological signs, severe respiratory distress and mortality. Nevertheless, the pathogenesis of EHDV-8 in this species remains poorly understood. This study aimed to evaluate the clinical and pathological impact of EHD in red deer, characterising clinical signs, main lesions, tissue distribution and target cells of EHDV. Clinical data were collected through a nationwide online survey and field outbreak studies, and histopathological and immunohistochemical analyses were performed on 16 confirmed EHDV-8 cases. Frequent clinical signs included neurological signs, ptyalism, lameness and dyspnoea. During the outbreak, average morbidity and mortality in captive red deer were 6.9% and 4.9%, respectively, with a case fatality rate of 70.3%, higher in males and almost exclusively in adults. Gross examination revealed extensive vascular and epithelial lesions with generalised congestion, focal to multifocal haemorrhages and marked pulmonary oedema, confirmed histologically with severe cellular damage. The most affected organs were the brain, lungs, liver, kidneys and spleen, showing interstitial oedema, vasculitis, mononuclear infiltration with necrosis, severe lymphoid depletion and gliosis with satellitosis in the brain. The EHDV antigen was primarily detected in macrophages, reticular cells, endothelial cells and epithelial cells. This study provides the first description of EHDV’s clinical and pathological impact in red deer, confirming their high susceptibility to EHDV-8.

**Supplementary Information:**

The online version contains supplementary material available at 10.1186/s13567-026-01807-w.

## Introduction

Epizootic haemorrhagic disease (EHD) is a viral disease caused by *Orbivirus ruminantium* (EHDV; Family *Sedoreoviridae*) which is closely related to bluetongue virus (BTV) [1]. EHDV is transmitted by biting midges of the genus *Culicoides* spp. [2]. The virus was originally described in North America in 1955, as a lethal disease in white-tailed deer (*Odocoileus virginianus)* and affects different domestic and wild ruminants [3, 4]. EHDV is a double-stranded RNA virus whose RNA segments encode seven structural (VP1–VP7) and at least four non-structural (NS1, NS2, NS3/NS3A, NS4) proteins [5]. To date, seven EHDV serotypes have been described (1–2 and 4–8), whose distribution varies heterogeneously across geographical regions.

EHDV-serotype 1 (EHDV-1), EHDV-2 and EHDV-6 are enzootic in North America, primarily affecting white-tailed deer [6]. Infections with these serotypes are characterised by high morbidity and mortality and include a wide range of clinical signs such as fever, weakness, inappetence, excessive salivation, facial oedema, hyperaemia of the conjunctiva and oral mucous membranes, as well as coronitis and stomatitis [7–10]. In cattle (*Bos taurus*), most of the EHDV-infected animals remain asymptomatic or experience transient mild clinical signs [11, 12]. However, an outstanding deviation from the typically asymptomatic EHDV infection in cattle is the Ibaraki strain of EHDV-2 in Japan, which has intermittently caused disease outbreaks since 1959 [13]. EHDV-6 was also implicated in multiple clinical outbreaks in cattle in Turkey and in North Africa [14]. In Asia, all EHDV serotypes have been documented, including a notable EHDV-7 outbreak in cattle in Israel that was primarily associated with a decrease in milk production [15]. EHDV-8 was first reported in cattle in Australia in 1982 [16]. After its initial identification, EHDV-8 was not reported again in association with clinical disease for several decades, and no further isolations were documented during this period. In 2021, EHDV-8 was associated with outbreaks in cattle [17] and Barbary deer (*Cervus elaphus barbarus*) [18] in Tunisia. However, phylogenetic analyses indicated that the EHDV-8 Tunisian strains detected in these outbreaks were genetically distinct from the historical Australian isolate [17]. Historically absent from Europe, the first EHD cases were confirmed in November 2022 in cattle in Sicily and Sardinia, Italy [19], and in cattle and red deer in southwestern Spain, both associated with EHDV-8 [4, 20]. Subsequently, in 2023, EHDV-8 spread rapidly northwards throughout the Iberian Peninsula affecting both domestic and wild ruminants [21]. By the end of 2023, the entire Spanish mainland was considered affected by EHD, reaching Portugal and France the same year [22]. Despite this rapid expansion of EHDV-8 in Europe, there is still a significant knowledge gap regarding its detailed pathological characterisation, especially in wildlife.

In Spain, a total of 267 EHD outbreaks caused by EHDV-8 were officially confirmed, affecting 263 of 482 (54.6%) local veterinary units across 15 of the 17 Spanish autonomous communities in 2023 [22]. These data represent an underestimation since cases were no longer required to be confirmed once the county was already declared positive for the disease. The EHDV-8 infected cattle typically presented self-limiting clinical symptoms [23], characterised by anorexia, fever, prostration, apathy, ptyalism, erythema and ulcers on the oral mucosa of the muzzle region and on udder, as well as respiratory distress, haemorrhagic diarrhoea, reduced milk production and, in some cases, reproductive sequelae including abortions and stillbirths [24]. Regarding wild ruminants, particularly red deer (*Cervus elaphus*), pathogenicity appears to be more severe than in cattle, with neurological signs including ataxia, loss of flight instinct and haemorrhagic lesions [20]. Although the data may be likely underestimated, 176 red deer deaths attributed to EHDV-8 were reported to World Organisation for Animal Health (WOAH) between July and December 2023 [22], as well as fatal cases in other free-ranging and captive wild species from the Bovidae and Cervidae families [25]. In addition to its clinical effects and possible impacts on biodiversity, the restrictions on the movement of live ruminants to other EU Member States, imposed under Delegated EU Regulation 2023/2515, led to significant economic losses.

These findings suggest higher pathogenicity of EHDV-8 in red deer than in cattle. However, the clinical presentation and pathological impact in red deer remain unknown. In the present study, we evaluated clinical signs, pathological lesions, tissue distribution and target cells of the virus in red deer naturally infected with EHDV-8.

## Materials and methods

### Clinical impact assessment

#### National surveys

Between November 2023 and April 2024, an anonymous online survey to better understand the clinical impact of EHDV-8 on red deer. The survey was created using Microsoft Forms® (Microsoft Corporation, Redmond, WA, USA) and consisted of 22 questions (Additional file 1) and collected information on several aspects, including farm or hunting-ground characteristics, the main clinical signs observed, the temporal distribution of cases, average duration of the outbreaks and affected animals by age. The questionnaire was developed and subsequently piloted with a panel of ten experts in game management and wildlife diseases to refine its content and ensure clarity and relevance prior to distribution. The survey targeted hunting estate managers, gamekeepers, veterinarians, hunters and farm owners in Spain and relied on voluntary participation. To ensure nationwide coverage, the survey was distributed through multiple channels, including veterinary professional colleges, hunting federations, digital platforms and social medial profiles of the involved institutions. Additionally, visual and informative visual posters were created to aid dissemination. As the survey was disseminated broadly without a defined sampling frame, a response rate could not be calculated.

#### Case study: EHD outbreak in farmed red deer

Between 15 October and 10 December 2023, an outbreak of EHD was monitored on a red deer farm in southern Spain comprising 1709 animals, including 933 males (712 adults and 221 juveniles < 1 year old) and 776 females (557 adults and 219 juveniles < 1 year old), distributed across 45 plots. Throughout this period, animals were systematically observed for clinical signs consistent with EHD. Clinical cases, treatments administered to affected individuals and mortality events were recorded. Data from symptomatic and deceased animals were stratified by sex and age group to identify potential epidemiological patterns and demographic differences in disease manifestation and outcome.

### Pathological evaluation

#### Study design

From mid‐July 2023 to August 2024, carcasses of 16 red deer (15 adult males, 1 juvenile male) found dead with macroscopic lesions compatible with EHD were collected from different provinces of Andalusia (southern Spain), Castile-La Mancha (central Spain) and Catalonia (northeastern Spain), while numerous outbreaks associated to EHDV-8 occurred in both wild and domestic ruminants [21]. Necropsies were conducted in the field or, when possible, at the Faculty of Veterinary Medicine of Córdoba [University of Córdoba (UCO), Spain], National Wildlife Research Institute (IREC; Ciudad Real, Spain) or the Faculty of Veterinary Medicine of Barcelona [Autonomous University of Barcelona (UAB), Spain], evaluating macroscopic lesions consistent with EHD. A systematic necropsy was performed on each animal following a standardised protocol when conducted in the necropsy rooms of the institutions, but not for necropsies performed in the field. In all cases included in this study, a systematic evaluation of macroscopic lesions was carried out, along with the collection of tissue samples (brain, heart, lung, liver, spleen and kidney) for subsequent histopathological and immunohistochemical analyses. The selection criteria for including the animals were the confirmation of EHDV-8 infection by molecular analyses in blood and/or spleen samples, as well as adequate carcass preservation for post-mortem evaluation. In parallel, lung, spleen, liver, kidney and heart samples were routinely cultured to rule out concurrent bacterial infection. Field conditions occasionally restricted the sampling of certain organs, most notably the brain.

#### Pathological study of the main affected organs

Tissue samples for histopathological and immunohistochemical studies were fixed in 10% neutral buffered formalin for 24 h, dehydrated through a graded series of ethanol, immersed in xylol and embedded in paraffin wax, using an automatic processor. Sections were cut at 3 μm and stained with haematoxylin–eosin (HE) using standard procedures to carry out the histopathological analysis. A semiquantitative evaluation of the severity of histopathological lesions in the brain, heart, lung, liver, spleen and kidney was performed including vascular and cellular alterations. Pathology scores were assigned follows: no significant lesions (0/−), mild (1/+), moderate (2/++), severe (3/+ ++) and very severe (4/++++). The evaluation of the lesions was carried out by three experienced observers.

### Molecular analysis for confirmation of EHDV-8 infection

Blood or spleen samples from the 16 red deer found dead were stored at −80 °C and analysed at the Animal Health laboratory of the UCO to confirm EHDV-8 infection. Additionally, samples from the brain, trachea, lung, heart, liver, kidney, lymph nodes and gonads (ovary/testis) were collected from four animals for further molecular analyses. Total RNA was extracted from post-mortem blood or spleen samples using the IndiSpin Pathogen Kit (Indical Bioscience, Leipzig, Germany) according to the manufacturer’s instructions. Amplifications were performed using the AgPath One-Step Real-Time Polymerase Chain Reaction (RT-PCR) Kit (Thermo Fisher Scientific, Massachusetts, USA) on a CFX Connect Real-Time Polymerase Chain Reaction (PCR) Detection System (Bio-Rad, CA, USA). A laboratory isolate of EHDV-8 obtained from a clinical sample collected in 2022 at the Spanish Central Veterinary Laboratory (the national reference laboratory for EHDV in Spain) was used as the positive control. Samples were initially subjected to a Pan-EHDV RT-Quantitative PCR (qPCR) targeting a 70 base-pair (bp) fragment of Segment 9 (Seg-9) [26]. An endogenous control targeting β-actin gen was included in each reaction. Pan-EHDV RT-qPCR positive samples were subsequently analysed using a serotype-specific RT-qPCR targeting a 173 bp fragment of EHDV-8 Seg-2 as previously described [27]. Thus, RNA denaturation heating at 95 °C for 3 min, 5 μL of denaturated RNA was added to the mix and the amplifications were carried out using the same cycling parameters for all of the assays: 45 °C for 10 min and 95 °C for 10 min followed by 40 cycles of 95 °C for 15 s and 60 °C for 1 min. Cycle threshold (Ct) values were recorded at the point when fluorescence crossed the predefined threshold; samples were considered positive when Ct $\le 37$, while negative samples were reported as "No Ct" for both RT-qPCR protocols. To exclude BTV infection, a Pan-BTV-Seg10-RT-qPCR was performed following the WOAH Terrestrial Manual [28].

### Monoclonal antibodies against VP7 EHDV

Monoclonal antibody (MoAb) 1B4B6 specific to recombinant VP7 (rec-VP7) was kindly provided by the Istituto Zooprofilattico Sperimentale dell’Abruzzo e del Molise ‘G. Caporale’ (IZSA&M) (Teramo, Italy) and produced as previously described [29]. Briefly, BALB/c mice (*n* = 4, 6–8 weeks old) were immunised intraperitoneally with a baculovirus-expressed rec-VP7-EHDV-2 antigen emulsified in Freund’s adjuvant, followed by two boosters. Sera were screened by indirect enzyme-linked immunosorbent assay (i-ELISA), and splenocytes from high-titre mice were fused with Sp2/O-Ag-14 myeloma cells. Hybridomas were cultured, screened by i-ELISA and subcloned by limiting dilution. MoAb cross-reactivity was assessed by i-ELISA against EHDV-2, BTV-2 and AHSV-1. Enzyme-linked immunosorbent assay (ELISA) detection was performed using horseradish peroxidase (HRP)-conjugated anti-mouse IgG and 3,3',5,5'-tetramethylbenzidine (TMB) substrate, with absorbance measured at 450 nm.

### Immunohistochemistry (IHC) for VP7 EHDV

Immunohistochemical analysis was performed on all selected animal tissues using the avidin–biotin-peroxidase complex (ABC) as previously described [30] with specific modifications to detect EHDV. To optimise the protocol, tissue samples were subjected to different unmasking methods for antigen retrieval or increasing permeability subsequently testing different concentrations of the primary MoAb 1B4B6. Details of the dilutions of the MoAb and pretreatments tested in this study, as well as an assessment of immunoreactivity by application of a subjective grading system for the intensity of the specific reaction are summarised in Table 1. Once the definitive protocol was chosen, it was used for the analysis of all the selected organs. Briefly, tissue sections (3 µm) were dewaxed and rehydrated. Endogenous peroxidase activity was exhausted by incubation with 0.3% hydrogen peroxide in methanol for 30 min at room temperature. To facilitate antigen exposure and improve tissue permeability, the optimum pretreatment was the incubation with proteinase K for 8 min at 37 °C in an oven. Subsequently, sections were rinsed three times in phosphate-buffered saline (PBS; pH 7.2) for 10 min, covered with 10% normal goat serum (Termo Fisher Scientific, Massachusetts, USA) in 0.05 M Tris buffered saline (TBS) (pH 7.6) for 30 min at room temperature and incubated with the 1B4B6 against the structural protein VP7 of EHDV (IZSA&M) overnight at 4 °C. After primary incubation, slides were washed in PBS (three times for 5 min each) and then incubated for 30 min at room temperature with a biotinylated goat anti-mouse IgG secondary antibody (Dako, Glostrup, Denmark) diluted 1:100 in TBS containing normal goat serum 10%. After three further 5 min washes in PBS, samples were incubated with the ABC complex (Vectastain^®^ ABC Elite Kit, Vector Laboratories, CA, USA) for 1 h at room temperature. All tissue sections were rinsed in TBS and incubated with the chromogen solution (NovaRED^®^ Substrate Kit, Vector Laboratories). Finally, slides were counterstained with Harris’ haematoxylin.

**Table 1:** **Immunolabelling of monoclonal antibody (MoAb) 1B4B6 against VP7 EHDV with different unmasking methods**

*Pretreatment*	*Time of pretreatment*	*VP7 EHDV antibody dilution*		
		1:50	1:500	1:1000
Proteinase K^a^	8 min	**+**	−	−
Citrate microwave (pH 6)^b^	6 min	Bs	−	−
Citrate autoclave (pH 6)^c^	10 min	**#**	−	−

^a^Incubation with 0.2% proteinase K (Sigma-Aldrich Chemie, Buchs, Switzerland) in Tris buffer for 8 min (min) at 37 °C in oven

^b^Incubation with 0.1 M tri-sodium citrate dihydrate (pH 6), microwave for 6 min at sub-boiling temperature

^c^Incubation with 0.1 M tri-sodium citrate dihydrate (pH6), autoclaved for 10 min at 121 °C and 1 atmosphere

Intensity of immunolabelling was scored as: none (−); positive reaction and light background (+); positive reaction but intense background (Bs); positive reaction but deteriorated tissue (#).

Positive controls used were spleen sections from an EHDV-8 RT-qPCR positive red deer with low Ct values (19–22). Negative control consisted of spleen sections from EHDV-negative red deer by RT-qPCR. Additionally, to confirm specificity, the primary antibody was replaced by mouse non-immune serum (Dako/Agilent, Glostrup, Denmark) on tissue sections from EHDV-infected red deer.

An immunohistochemical evaluation was performed to detect VP7-positive target cells in the different organs of the animals evaluated in the section “Pathological evaluation”. The evaluation of the viral antigen distribution was carried out by two experienced observers.

### Statistical analysis

To assess differences in morbidity and mortality rates according to sex and age class, statistical analyses were performed using the chi-squared (*χ*^2^) test for categorical variables, and *p*-values were calculated to assess statistical significance. Analyses were conducted using SPSS statistical software, version 29.0.2.0 (Statistical Package for Social Sciences Inc., Chicago, IL, USA). For the survey data, a descriptive analysis was carried out, and differences in perceived incidence and mortality between groups were assessed using a Poisson test for count data. Confidence intervals for mean counts were calculated assuming a Poisson distribution. The prevalence of histopathological lesions or EHDV detection in the different tissues was determined as the proportion of positive animals relative to the total number examined, using the Epitools epidemiological calculator (version 0.5–10; Ausvet Pty Ltd, Australia). Histopathological evaluations and graphs were generated using total (summed) lesion scores and analysed with GraphPad Prism version 8 (GraphPad Software Inc.; San Diego, CA, USA). Differences in lesion scores among organs were assessed using the Kruskal–Wallis test. Statistical significance was set at *p* ≤ 0.05.

### Ethics approval

For the surveys, the bioethics evaluation of the protocol was approved by the Ethics Committee of the UCO (reference ETICA-UCO-058–2023), following the provisions of current legal regulations and, in particular of the Law 3/2018 on the Protection of Personal Data and guarantee of digital rights. The pathological study did not involve purposeful killing of animals, as we only employed animals dying during a disease outbreak, so no ethical evaluation was needed according to the Spanish and EU experimental animal protection law. Animal sampling was performed by qualified veterinarians in compliance with the Ethical Principles in Animal Research.

## Results

### Clinical impact assessment

**Figure 1:**
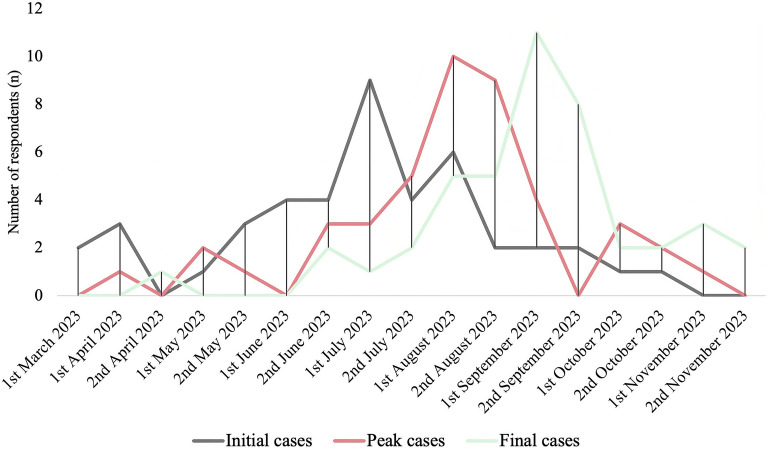
**Seasonality of red deer** (***Cervus elaphus***) ** EHD outbreaks in Spain from March to November 2023, based on data from 48 nationwide hunting estate surveys**. The analysis was divided into three periods – initial cases, peak cases and final cases – with data grouped at 15-day intervals. The *y*-axis represents the number of respondents (*n*).

**Figure 2:**
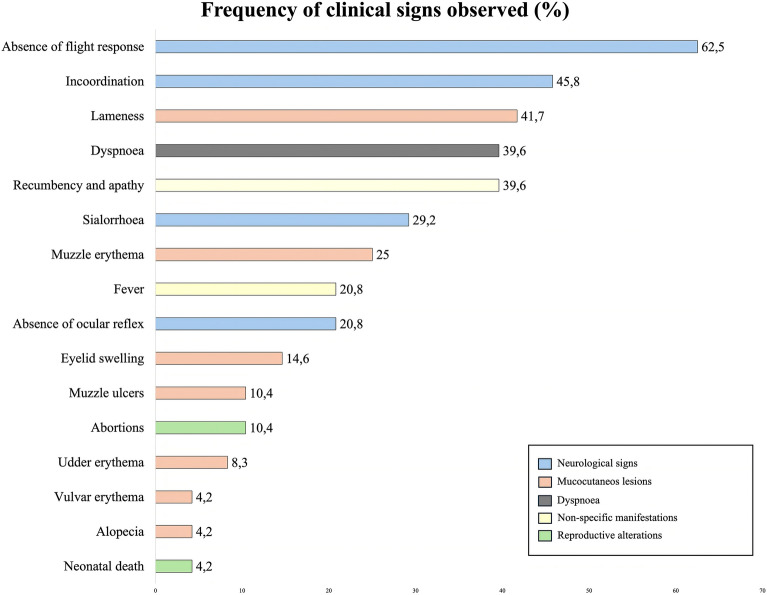
**Frequency of clinical signs observed in red deer **(***Cervus elaphus***)** affected by EHD, based on 48 nationwide surveys conducted between March and November 2023 in Spain**. Percentages indicate the proportion of hunting estates in which animals with the indicated clinical sign were detected.

The survey was completed by a voluntary sample of professionals related to wildlife, such as environmental wildlife wardens (8.7%), game estate owners (11.0%), veterinarians (13.0%), gamekeepers (15.2%), engineers (15.2%) and other professionals (36.9%) representing 48 hunting estates across 17 different provinces in Spain. The mean surface area of these estates was 4613 ha (range: 1007–4000 ha). The proportion of surveyed estates with high-wire perimeter fencing (54.2%) did not differ significantly from those without it (45.8%). Reported cases in red deer were most frequently described in the surveys as occurring between June and October 2023, although three suspected cases were recorded in March 2023 (Figure 1). Reported case occurrence appeared to peak in August and declined thereafter, with the last cases recorded in November 2023, according to the surveys. The estimated average duration of reported EHD outbreaks in the surveyed hunting estates was 64 days, with an average time from onset to peak of 34 days and from peak to the cessation of clinical signs of 32 days. Respondents described the clinical presentation in red deer as mainly acute or hyperacute, with neurological signs in 20.8–62.5% of cases and dyspnoea in 39.6% being the most frequent (Figure 2). The lack of reliable hunting estate-level censuses limited our ability to calculate morbidity, mortality and case-fatality rates associated with EHD in free ranging red deer. Nonetheless, respondents reported a significantly higher perceived incidence of clinical EHD in adult red deer [mean: 66.5, 95% confidence interval (CI) 62.3–70.6] than in juveniles (mean: 13.6, 95% CI 11.7–15.5), with confidence intervals calculated assuming a Poisson distribution, reflecting reported frequencies within the survey responses (Poisson test, *z* = 22.88, *p* < 0.001). In addition, respondents estimated the mean number of adult red deer deaths per hunting estate resulting from EHD to be 12.3 males (95% CI 10.9–13.7) and 25.8 females (95% CI 23.9–27.8), indicating a significantly higher reported number of deaths in females (Poisson test, *z* = −33.21, *p* < 0.001).

**Figure 3:**
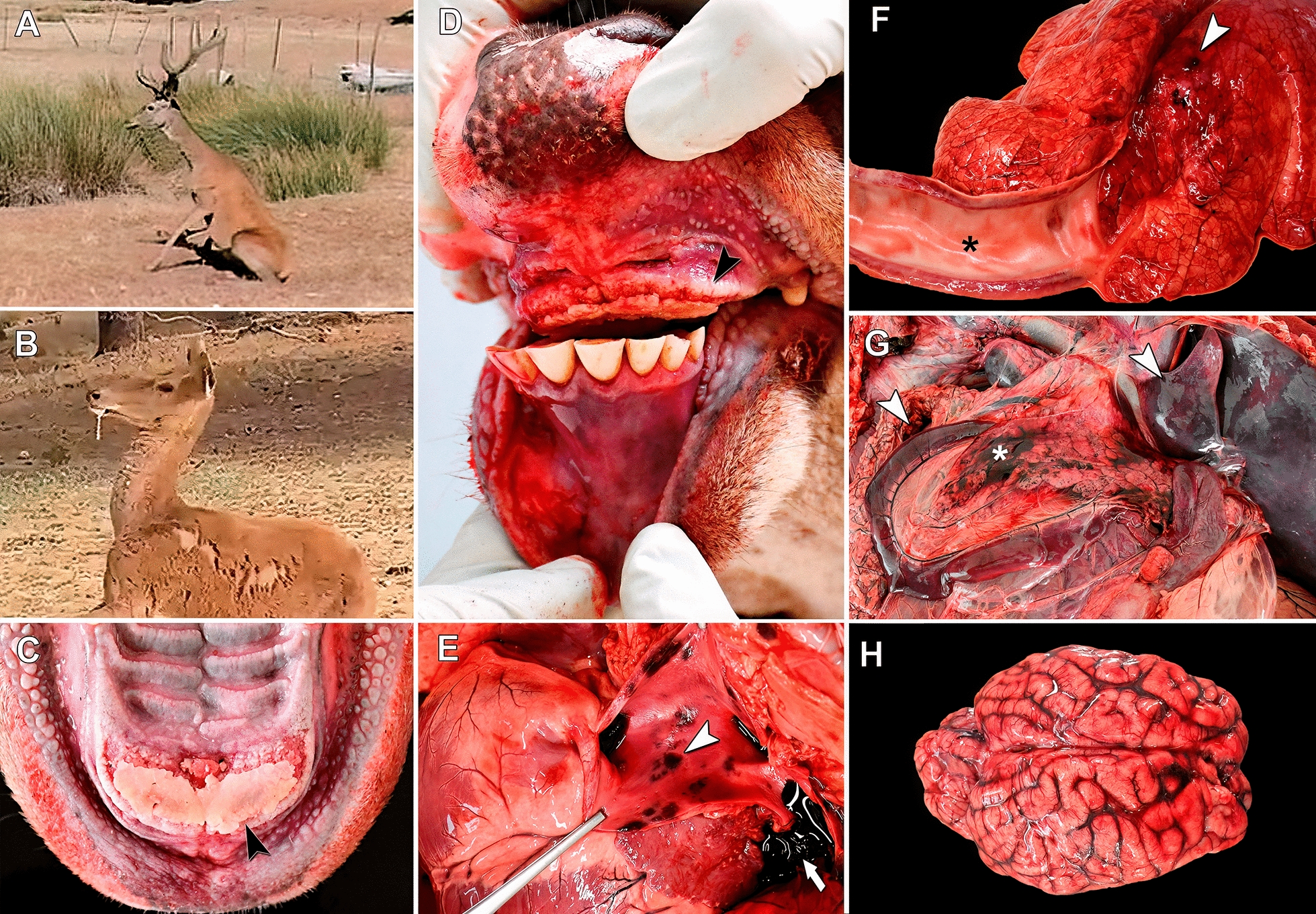
**Representative images of gross lesions in red deer** (***Cervus elaphus***) ** naturally infected with EHDV-8**. **A** EHDV-8-positive male red deer showing incoordination and ataxia. **B** EHDV-8-positive female red deer with opisthotonos and ptyalism. **C** Erosion and ulceration (black arrowhead) of the oral mucosa with necrosis of the dental pad. **D** Erosion and ulceration (black arrowhead) of the oral mucosa with necrosis of the dental pad and cyanosis of the oral mucosa. **E** Haemorrhages (white arrowhead) in the tunica media and intima of the vessel wall at the base of the pulmonary artery, accompanied by a lack of post-mortem blood coagulation (white arrow). **F** Lungs with congestion, multifocal petechiae (white arrowhead) and abundant foam in the trachea (black asterisk). **G** Severe congestion of the intestinal loops and liver (white arrowheads), along with pancreatic haemorrhages (white asterisk). **H** Brain and cerebellum with severe congestion.

During the outbreak at the red deer farm in southern Spain, animals exhibited a spectrum of neurological, respiratory and systemic alterations, which frequently culminated in rapid death, either in the absence of observable clinical signs or within a few hours to one day after the clinical onset. Neurological signs included ataxia (Figure 3A), repetitive head movements, opisthotonos and ptyalism (Figure 3B), recumbent pedalling, blindness with mydriasis and, in severe cases, coma. Behavioural changes such as prostration and isolation from the group were common. Fever, respiratory distress and occasional haematuria were also observed. Out of the 1709 animals on the farm, clinical signs were observed in 118 animals, yielding an overall apparent morbidity rate of 6.9% (118/1709). Morbidity was significantly higher in males (8.7%; 81/933) compared with females (4.8%; 37/776) (*χ*^2^ = 9.50, *p* = 0.002). Consequently, males comprised 68.6% (81/118) of all clinically affected animals. The clinical course was acute, with death occurring within hours or, at most, within 24 h after onset. Most fatal cases were found dead without preceding clinical signs. Overall mortality was 4.9% (83/1709). Consistent with morbidity trends, sex-specific mortality was significantly higher in males (6.3%; 59/933) than in females (3.1%; 24/776) (*χ*^2^ = 8.88, *p* = 0.0029), with males accounting for 71.1% (59/83) of all deceased animals. The overall case fatality rate was 70.3% (83/118); however, differences between sexes were not statistically significant (*χ*^2^ = 0.44, *p* = 0.51). Nearly all clinically affected animals (99.0%) were adults (> 3 years old), except for a single juvenile. When treatment was possible, affected animals received antibiotic-based therapies against clostridial coinfections (tetracycline, penicillin and cephalosporin), as well as non-steroidal anti-inflammatory drugs (NSAIDs). Following treatment, a total of 63.5% (33/52) these animals recovered. However, among the 13 surviving females, 1 developed fatal dystocia, whereas 10 aborted during the subsequent gestation. Additionally, deltamethrin pour-on was applied as an external antiparasitic treatment, and vaccination against *Clostridium perfringens* was undertaken.

### Pathological evaluation

#### Macroscopic findings

All 16 red deer included in the pathological study were found dead and exhibited macroscopic lesions consistent with EHD. In all cases, EHDV-8 infection was confirmed in blood or spleen samples by Pan-EHDV RT-qPCR and EHDV-8-specific RT-qPCR, and/or by IHC. In addition, all animals tested negative for BTV infection. Among the four animals subjected to bacterial culture, three yielded positive growth on *Clostridium*-selective TSN medium. Confirmation by RT-qPCR, performed at an external laboratory, identified three *Clostridium* species, including the detection of the *alpha*, *epsilon* and *beta*2 toxin genes of *Clostridium perfringens*.

External pathological examination frequently revealed cutaneous lacerations, erosions and superficial skin wounds, as well as a marked congestion of the oral mucosa, in some cases accompanied by partial detachment of the dental pad mucosa (Figure 3C, D). Necropsy findings indicated severe systemic vascular alterations, with the most prominent findings being generalised congestion and focal and multifocal haemorrhages, including the tunica intima and media of the pulmonary arterial wall (Figure 3E). One of the most consistent gross findings was pulmonary oedema, characterised by presence of abundant frothy fluid within the airways, as well as enlarged, heavy and wet lungs, with interlobular septa widened by oedematous fluid (Figure 3F). Widespread petechial haemorrhages were also observed. In many animals, the absence of post-mortem blood coagulation was noted, together with haematuria and petechiae on the mucosa of the urinary bladder and haemorrhagic enteritis (Figure 3G), indicating extensive vascular leakage and mucosal damage in tubular organs. The brain exhibited meningeal congestion (Figure 3H). These alterations were especially evident in animals with more severe clinical signs and rapid disease progression.

#### Histopathological findings

**Figure 4:**
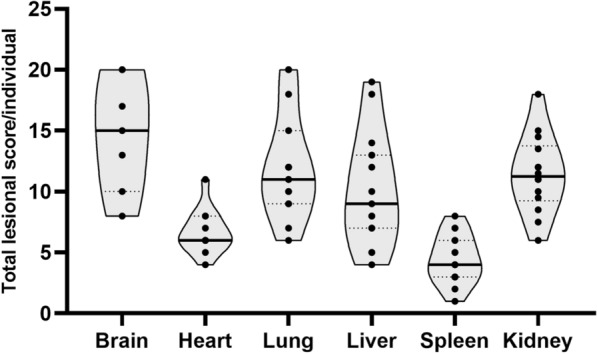
**Distribution of total lesional scores per individual** **(*n*** **= 16) across internal organs**. Violin plots show the total lesional score (sum) per animal in the brain, heart, lung, liver, spleen and kidney. Each dot represents an individual animal, solid lines indicate the median, and dotted lines represent the interquartile range

Microscopic examination revealed multi-organ involvement in animals infected with EHDV-8, with lesion severity varying across tissues. The main histopathological lesions identified in the examined organs and included in the semiquantitative scoring are summarised in Table 2 and illustrated in Figure 4. Semiquantitative scoring was restricted to organs yielding an adequate sample size (brain, heart, lung, liver, spleen and kidney). Significant differences in lesion severity were detected among evaluated organs (*p* < 0.05), reflecting distinct local host–pathogen interactions. Notably, the brain, lung and kidney were the most severely affected tissues, exhibiting the highest lesion burdens. All other tissues were also examined qualitatively whenever samples were present. In cases where certain tissues could not be assessed, this was due either to the absence of suitable samples or advanced autolysis.

**Table 2:** **Average histopathological lesions in the main affected organs of red deer (*Cervus elaphus*) naturally infected with EHDV-8**

Lesion type	Brain (*n* = 7)	Heart (*n* = 13)	Lung (*n* = 15)	Liver (*n* = 13)	Spleen (*n* = 16)	Kidney (*n* = 14)
Vascular changes						
Congestion	+ +	++	+++	++	++	++
Haemorrhage	+	++	+++	+	+	++
Oedema	++	++	++	+	−	−
Vasculitis	++	++	+	++	−	−
Inflammatory and necrotic changes						
Mononuclear infiltrate	−	+	++	++	−	++
Necrosis	++	+	+	+	++	++
Organ-specific changes						
Gliosis	+++					
Satellitosis	+++					
Demyelination/spongiosis	+					
Emphysema			++			
Atelectasis			+			
Bronchiolar epithelium desquamation			+			
Kupffer cell proliferation				+		
Vacuolar degeneration				+		
Lymphoid depletion					++	
Macrophage/phagocytic cell hyperplasia					+	
Tubular degeneration						+++
Renal casts						++
Proliferative glomerulonephritis						+

Severity of lesions was scored as: no significant lesions (−); mild (+); moderate (++) and severe (+++).

**Figure 5:**
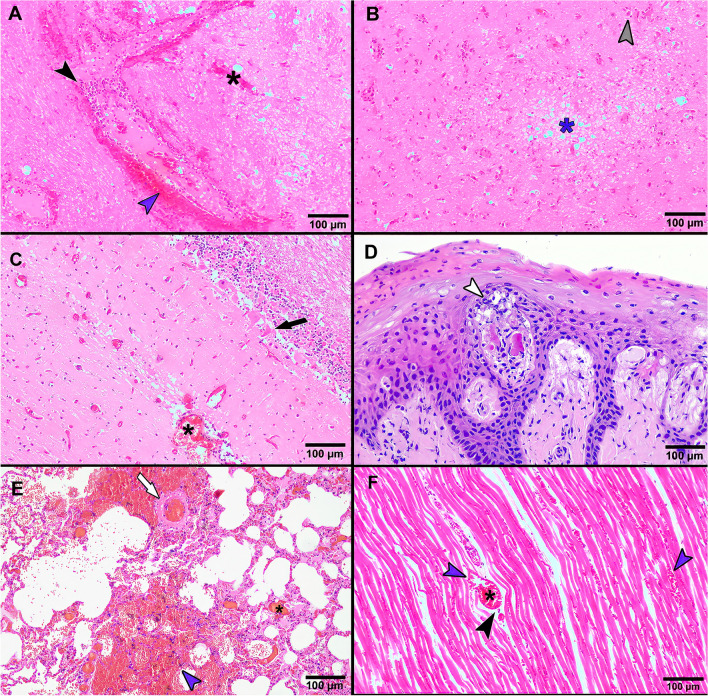
**Main histopathological findings from 16 red deer** (***Cervus elaphus***)** found dead and confirmed to be infected with EHDV-8**. **A** Brain showing congestion (black asterisk), haemorrhages (blue arrowhead) and perivascular cuffing (black arrowhead). **B** Brain presenting extensive gliosis and satellitosis (grey arrowhead), together with parenchyma necrosis (blue asterisk). **C** Cerebellum showing congestion (black asterisk) and necrosis of Purkinje cells (black arrow). **D** Oral mucosa showing well-demarcated intraepidermal pustules (white arrowhead) expanding the stratum spinosum, composed predominantly of degenerate neutrophils admixed with cellular debris **E** Lung tissue exhibiting severe congestion (black asterisk) and haemorrhages (blue arrowhead), together with marked thickening of the vascular wall (white arrow). **F** Heart showing congestion (black asterisk), haemorrhages (blue arrowhead) and perivascular oedema (black arrowhead). Staining: haematoxylin–eosin.

*Central nervous system*: The brain was the most consistently and severely affected organ (Figure 4). Congestion, widespread perivascular oedema and neuronal swelling together with extensive gliosis, satellitosis and neuronal necrosis were detected in 100% (7/7) of the evaluated animals, generally with moderate severity (Table 2; Figure 5A, B). Perivascular cuffs composed of lymphocytes/mononuclear cells were also observed in 85.7% (6/7) of animals in the brain parenchyma and were of moderate severity (Table 2; Figure 5A). Less frequent alterations included multifocal areas of demyelination, axonal spheroids and spongiosis, found in 28.6% (2/7) and 42.9% (3/7) of animals, respectively, as well as haemorrhages (Figure 5A), present in 42.9% (3/7), predominantly mild (Table 2). In addition, the meninges frequently showed inflammatory involvement, characterised by oedema, vascular congestion and haemorrhages. In the cerebellum, in addition to the previously described lesions, occasional necrosis of Purkinje cells was observed (Figure 5C).

*Oral mucosa*: The oral mucosa of the muzzle exhibited vascular congestion accompanied by a moderate mononuclear inflammatory infiltrate within the lamina propria and well-demarcated intraepidermal pustules composed predominantly of degenerate neutrophils admixed with cellular debris (Figure 5D).

*Respiratory system*: The lungs were one of the most consistently affected organs (Figure 4). Lung tissue showed pronounced and widespread vascular and inflammatory alterations. Severe congestion together with interstitial and alveolar haemorrhages were present in the 100% of the analysed red deer (15/15) (Figure 5E), while moderate interstitial oedema was detected in 93.3% (14/15) of animals. Mild alveolar oedema was observed in 46.7% (7/15) of the individuals. Mild to moderate vasculitis with mononuclear inflammatory infiltration and endothelial swelling was identified in 66.7% (10/15) of the lungs examined (Figure 5E; Table 2). A mononuclear interstitial infiltrate affecting the pulmonary parenchyma was also observed in 73.3% (11/15) of animals, consistent with interstitial pneumonia. Necrotic foci involving both endothelial and interstitial cells were present in 46.7% (7/15) of samples. Other finding included emphysema (86.7%; 13/15), atelectasis (33.3%; 5/15) and bronchiolar epithelial desquamation (33.3%; 5/15). In addition, some animals exhibited lesions consistent with chronic processes, including fibrosis in the interlobular septa, parenchyma and pleura, angiogenesis and the presence of lung nematodes. In the trachea, the most consistent lesions included submucosal congestion and haemorrhages, frequently accompanied by mononuclear inflammatory infiltrates and desquamation of the respiratory epithelium.

*Heart*: Cardiac tissue showed generally milder and less consistent lesions compared with the other organs evaluated (Figure 4). The most common findings were moderate vascular alterations, with congestion and myocardial haemorrhages observed in 100% (13/13) of animals, and perivascular oedema present in 92.3% (12/13) (Figure 5F). At the cellular level, mononuclear inflammatory infiltrates were detected in 84.6% (11/13) of cases, with mild to moderate severity (Table 2). Myocardial necrosis with a multifocal distribution pattern was observed in 46.2% (6/13) of animals and was generally mild (Table 2).

**Figure 6:**
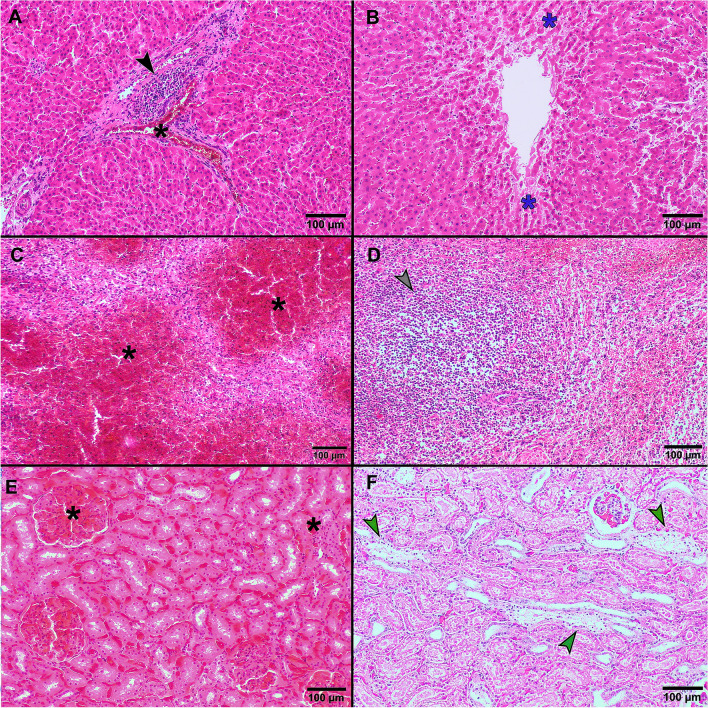
**Histopathological findings in red deer **(***Cervus elaphus***)** naturally infected with EHDV-8**. **A** Liver showing portal congestion (black asterisk) and perivascular cuffing (black arrowhead). **B** Liver with centrilobular necrosis (blue asterisks). **C** Spleen presenting vascular changes (black asterisk) and **D** a prominent lymphoid depletion (grey arrowhead). **E** Kidney with severe congestion at glomerulus and peritubular capillaries (black asterisk) and **F** tubular necrosis (green arrowheads).

*Liver* **–** Hepatic lesions were frequent and heterogeneous. Vascular alterations were notable in this organ, with sinusoidal congestion observed in 100% (13/13) of animals and moderate to severe portal congestion present in 71.4% (9/13) (Figure 6A; Table 2). Moderate mononuclear infiltrates with a multifocal distribution pattern were common in both the hepatic parenchyma (64.3%; 8/13) and periportal regions (92.9%; 12/13) (Figure 6A). Centrilobular hepatocellular coagulative necrosis was detected in 85.7% of individuals (11/13) (Figure 6B), generally with moderate severity, while mild endothelial necrosis was observed in the centrilobular vein in 35.7% of the animals (5/13). Other less frequent findings included Kupffer cell proliferation, vacuolar cytoplasmic hepatocellular degeneration and focally extensive neutrophilic infiltrates in Glisson’s capsule, which were observed in a limited number of cases.

*Lymphoid system*: In the spleen, moderate congestion (Figure 6C) and necrotic changes were reported in 62.5% (10/16) and 81.3% (13/16) of individuals, respectively, with marked lymphoid depletion (Figure 6D and Table 2) present in 100% (16/16) of the animals. Macrophage-phagocytic cell hyperplasia, as well as dendritic and trabecular cell proliferation were sporadic findings. Lymph nodes showed vascular alterations, including generalised congestion, medullary haemorrhages and perivascular oedema, along with prominent lymphoid depletion, as described previously in the spleen.

*Kidney*: Congestion (100%; 14/14) and haemorrhages (92.6%; 13/14) were commonly observed throughout the renal parenchyma (Figure 6E), with greater frequency and severity in the medulla compared with the cortex. Severe multifocal tubular degeneration and moderate interstitial mononuclear inflammatory infiltrates were present in all animals (100%; 14/14) and represented the most consistent parenchymal lesions. Tubular necrosis was observed in 85.7% (12/14) of specimens (Figure 6F), while acellular renal casts were present in 64.3% (9/14) of cases, generally with mild severity. Mild proliferative glomerulonephritis was also reported in some animals (42.9%; 6/14) (Table 2).

### Viral distribution in tissues

EHDV-8 was detected in the spleen of all animals analysed by RT-qPCR. Moreover, the presence of the virus was confirmed by this technique in the liver, kidney, heart, brain, lung, lymph nodes, trachea, gonads (ovary and testis) and oral mucosa of the muzzle region of the four animals from which complete set of tissues were obtained.

**Figure 7:**
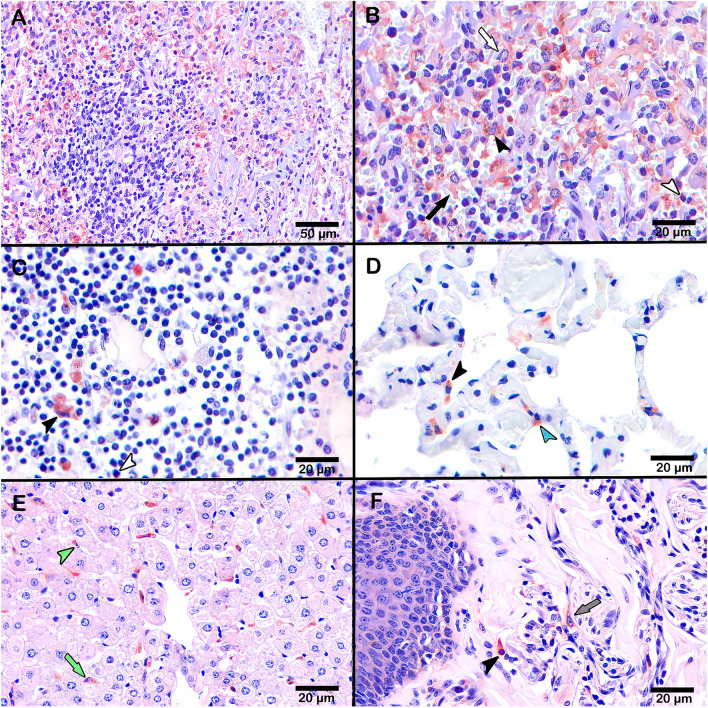
**Immunolabelling of cells with MoAb 1B4B6 against VP7 EHDV in red deer ** (***Cervus elaphus***) ** naturally infected with EHDV-8**. **A** and **B** Spleen sections showing positivity in macrophages (black arrowhead), lymphocytes (white arrowhead), reticular (white arrow) and dendritic (black arrow) cells. **C** Lymph node section showing positivity in both lymphocytes (white arrowhead) and macrophages (black arrowhead) within the cortex. **D** Lung section showing positivity in epithelial cells (blue arrowhead) and macrophages (black arrowhead). **E** Liver section showing positivity in endothelial (green arrowhead) and Kupffer (green arrow) cells. **F** Section of oral mucosa from the muzzle region showing positivity in macrophages, some of which exhibit peripheral chromatin condensation (black arrowhead), and in dermal fibroblasts (grey arrow). Immunohistochemistry (ABC method).

The immunostaining against EHDV VP7 showed an intracytoplasmic granular red stain in infected cells. Viral antigen was primarily detected in lymphocytes and macrophages as well as in epithelial, endothelial and reticular cells. In the spleen, most immunopositive cells were located in the red pulp (Figure 7A), predominantly associated with macrophages and, to a lesser extent, with lymphocytes, endothelial, dendritic and reticular cells (Figure 7B). In lymph nodes, immunolabelling was primarily observed in both lymphocytes and macrophages within the cortex and medulla (Figure 7C). In the lungs, viral antigen was primarily observed in interstitial macrophages (Figure 7D), while in some cases it was also detected in other pulmonary sites, including bronchiolar epithelial cells and alveolar macrophages. In the liver, immunopositivity was mainly confined to the hepatic sinusoids, particularly in Kupffer and endothelial cells (Figure 7E). In the oral mucosa of the muzzle region, viral antigen was predominantly observed within the inflammatory infiltrates, composed mainly of macrophages, and in fibroblastic cells (Figure 7F). No immunopositivity was recorded by IHC in the brain, kidney, heart, trachea or tongue of any examined animal.

## Discussion

This study provides the first in-depth analysis of the clinical and pathological effects of natural EHDV infection in red deer. Through a combination of surveys, field outbreak studies, gross pathology, histopathology and immunohistochemistry, we characterised the clinical impact, evaluated the extent and severity of lesions across multiple organs, identified viral target cells and described the systemic pathological effects of the disease. This comprehensive approach also allows comparative analyses with pathological findings reported in other wild and domestic ruminant species and across different EHDV serotypes.

The surveys suggested a marked seasonality of EHD in Spain, with most red deer cases reported between July and October, coinciding with the main activity period of the most relevant *Culicoides* vectors in the country [31]. This pattern is consistent with previous findings on vector abundance seasonality [32] and with official reports of cases in cattle [21]. The mean duration of EHD outbreaks reported in the hunting states included in the surveys was 64 days, with a mean interval of 34 days from onset to the peak of clinical incidence. This temporal pattern is consistent with the close relationship between clinical signs and viraemia, aligning with previous reports describing a viraemia duration of approximately 3 weeks in naturally and experimentally infected white-tailed deer [33, 34]. However, the longer duration observed at the population level compared with the approximately 3-week viraemia reported in individual animals likely reflects the contrasting dynamics of experimental and natural infections. In experimental studies, all animals are typically exposed simultaneously, whereas under natural conditions transmission via *Culicoides* occurs over time, resulting in a broader infection window [35, 36]. Disease resolution in the surveys was reported to occur approximately 32 days after the peak of infection, a timeframe that could be explained by the induction of neutralising antibodies, which are consistently detectable by around 10–14 days post‑infection in white-tailed deer [37]. Evidence from field studies indicates that natural EHDV infection stimulates robust antibody responses in exposed animals, supporting clinical recovery and reducing population susceptibility to subsequent infections [38].

The clinical presentation of EHD in red deer was predominantly acute or hyperacute, consistent with observations from the first confirmed cases in red deer in Spain [20]. This is also in line with the acute and hyperacute disease forms observed in white-tailed deer infected with EHDV-1 and EHDV-2, the serotypes mostly associated with clinical cases in North America [3]. The rapid progression of the disease sometimes made detection prior to death difficult. Epidemiological indicators from EHD outbreaks are scarce in the literature. In cattle, reported morbidity rates vary widely from 0% to 13% in infections with EHDV-2 [13, 39] to 5–80% in infections with EHDV-7 [40]. In our study, red deer showed an apparent morbidity of 6.9%, a value within the lower range of those described in cattle. In free-ranging cervids, disease detection is challenging, and available data are limited, likely leading to an underestimation of the true impact of EHD. The overall mortality was estimated at 4.9% on the basis of field observations, suggesting moderate population-level mortality but a high case-fatality rate (70.9%) among clinically affected individuals. This pattern is consistent with reports from other highly susceptible species, with an estimated 67.0% case-fatality rate in infected white-tailed deer during field outbreaks [9] and similar values reported in experimental infections [41]. Results from both our national surveys and the farm outbreak investigation indicated that EHD seems to have a greater impact on adult red deer compared with juveniles. This pattern aligns with experimental studies of EHDV-2 infection in white-tailed deer, in which clinical disease was moderate in juveniles and no mortality was reported [42]. Sex-specific analyses revealed higher mortality and case-fatality rates in males compared with females. This heightened susceptibility may have been facilitated by the severe physiological stress and poor body condition experienced during the autumn rutting season, when adult individuals drastically reduce food intake [43]. However, estimates derived from the national surveys, which reported a mean number of 12.3 adult males and 25.8 adult females found dead, appear inconsistent with our findings in the affected red deer farm. This apparent divergence may be explained by differences in population structure between female-biased free-ranging deer populations and male-biased farmed deer systems [44]. Moreover, it should be noted that survey-derived findings reflect reported perceptions from respondents, whereas field and outbreak investigation data provide direct observational evidence.

Clinical manifestations of EHDV infection vary by host species and serotype. Likewise, although all organs were examined with the same level of detail, organ-specific lesion categories were only identified in the brain, lung and kidney, reflecting a greater diversity of histopathological changes in these tissues.

Data from EHDV-8-farmed-infected red deer, together with field reports from the national survey highlighted a predominance of neurological signs, with loss of the flight response and ataxia being among the most frequently observed manifestations in red deer. Neurological lesions were also particularly prominent in our pathological analysis, with the brain being the most consistently affected organ histologically. Gliosis, satellitosis, neuronal degeneration and nonsuppurative perivascular cuffing were consistently observed, in line with the pronounced neurotropism of EHDV-8 in red deer. This is further evidenced by RT-qPCR-positive results in the brains of all fully sampled animals (4/4). Although neurological signs and central nervous system lesions have been reported in wild ruminants infected with other EHDV serotypes, e.g., EHDV-2 infection in white-tailed deer, yak (*Bos grunniens*) and mule deer (*Odocoileus hemionus*) [45–47] and EHDV-6 infection in reindeer (*Rangifer tarandus*) [48], these manifestations are uncommon and generally less pronounced than those observed in the present study and in recent reports of EHDV-8 infection [49]. Neurological manifestations are also rare in cattle in which central nervous system involvement has not been documented regardless of serotype. Therefore, the extent and severity of EHDV-8-induced neurological alterations reported in this study are noteworthy and suggest that red deer may exhibit a heightened neuropathological susceptibility to EHDV infection.

Respiratory signs such as dyspnoea and nasal discharge, as well as lameness and mucosal erosions, were also reported in red deer infected with EHDV-8. These clinical manifestations were markedly more severe that those observed in cattle infected with the same serotype in which disease is typically subclinical or limited to mild clinical signs, including oral lesions, dysphagia, hoof lesions, tongue oedema, dehydration and dyspnoea [24]. Moreover, the frequent observation of pulmonary congestion, oedema and frothy serous fluid in the airways strongly suggests that acute respiratory distress is a prominent clinical feature in red deer, comparable to that reported in white-tailed deer [7, 10, 34], as well as in cattle affected by Ibaraki virus (EHDV-2) [50]. Both macroscopic and microscopic examinations consistently revealed that EHDV-8 is associated with a severe multisystemic disease in red deer, characterised by widespread vascular damage, including marked congestion, haemorrhages, extensive oedema, inflammation and tissue necrosis, particularly in the lungs. The coincidence of these lesions with the detection of EHDV antigen in interstitial macrophages and epithelial cells within the lung suggests that local viral replication and immune activation may contribute to endothelial damage and vascular leakage, ultimately leading to the haemorrhages, oedema and necrosis in this organ. These findings align with the pathological phenotype described in white-tailed deer [10, 34] and in cattle [51] infected with EHDV-6, where disruption of vascular integrity represents a key pathogenic mechanism in the EHD [7, 10, 52]. In addition, the histological analysis of cardiac lesions revealed myocardial congestion and intermyofibrillar haemorrhages accompanied by moderate interstitial mononuclear inflammatory infiltrates, findings consistent with systemic endothelial damage, aligning with previous descriptions in white-tailed deer infected with EHDV-2 and EHDV-6 [10, 45, 53]. The cardiovascular dysfunction observed in combination with respiratory compromise, plays a significant role in the pathophysiology of acute fatal outcomes in EHDV-8-infected red deer.

The liver also exhibited prominent vascular and inflammatory alterations, including sinusoidal congestion, haemorrhages and, in some cases, periportal mononuclear infiltrates. Sinusoidal congestion has been previously reported in white-tailed deer infected with EHDV‑1 and EHDV‑6 [7, 10, 34] and in Barbary deer infected with EHDV‑8 [18]. Notably, multiple areas of coagulative necrosis were observed, suggesting hepatocellular damage secondary to hypoxia and endothelial dysfunction, as previously described [8]. EHDV immunopositivity was observed particularly in Kupffer cells and endothelial cells, indicating that these hepatic lesions may result from both direct viral effects and in response to endothelial dysfunction [10]. These vascular and necrotic lesions were also evident in the spleen, together with marked lymphoid depletion, which was likewise observed in the studied lymph nodes. This depletion likely reflects the viral tropism for immune cells such as macrophages and lymphocytes, as reported in other *Orbivirus* infections [4, 54, 55], and may indicate immunosuppression during the acute phase of infection [56], resulting in a transient state that predisposes the host to opportunistic infections [57]. Moreover, the detection of EHDV antigen in these cells suggests their potential role in the dissemination of EHDV-8, as has been described in BTV-infected small ruminants, supporting their possible involvement in systemic spread, endothelial targeting and the pathogenesis of the associated vascular lesions [58].

Renal pathology was another prominent feature, with macroscopic evidence of friable, pulpy kidneys, even in animals necropsied shortly after death, as previously described [7]. Histologically, lesions included tubular degeneration, tubular epithelial necrosis, proliferative glomerulonephritis and vascular damage in both the cortical and medullary regions. These findings align with previous reports of EHDV-1,2,6 in white-tailed deer [7, 34, 46] and EHDV-2 in mule deer [46]. To the best of our knowledge, haematuria has not been specifically reported in previous cases of EHD. However, the severe renal and vascular pathology observed in red deer naturally infected with EHDV-8 may be consistent with the occurrence of haematuria described in the present study. Consequently, future research should determine whether the virus could be excreted in urine, as this could provide additional insight into its pathogenesis and potential routes of transmission. Furthermore, viral RNA was detected in the gonadal tissues of two males and two females, suggesting that EHDV may reach the reproductive organs in red deer. This finding could potentially explain reproductive disorders reported in cattle infected with EHDV‑6 [59] and EHDV-8 [24], although the associated reproductive consequences in red deer have not yet been fully investigated.

However, the use of naturally infected animals entails certain limitations, as it may introduce variability in the infectious dose, timing of exposure and stage of disease among the individuals studied. Moreover, there was a lack of uniformity in field necropsies, with sampling varying between animals in terms of the type and number of organs collected. This resulted in a limited number of complete tissues set available for IHC or molecular analysis. Additionally, limitations in IHC sensitivity for EHDV detection may have led to an underestimation of viral antigen distribution. Specifically, the MoAb used in this study had not previously been validated for IHC, and its reduced sensitivity may be attributable to several factors, including limited antibody affinity, antigen loss during fixation, suboptimal sampling time, or compromised tissue preservation, given that most outbreaks occurred during the high summer temperatures of southern Spain. Despite these constraints, the concordance between the clinical signs, gross and histological lesions and the detection of viral antigen in key tissues by RT-qPCR and IHC provides robust evidence of EHDV-8-induced pathology. Further experimental studies under controlled conditions are required to elucidate the temporal dynamics of lesion development, viral tissue tropism and host immune responses.

In conclusion, this study provides novel insights into the pathogenesis of EHDV-8 in a previously uncharacterised host and within a new geographical and epidemiological context. EHDV-8 is associated with a systemic and often severe disease in red deer, characterised by marked neurotropism, endothelial damage and multi-organ involvement. This contrasts with the generally milder presentation observed in cattle under the same epidemiological conditions [24]. In naturally infected red deer, EHDV-8 exhibits a widespread tissue distribution, which may be related to viral detection in macrophages and lymphocytes, resulting in lymphoid depletion. However, further experimental studies are needed to better elucidate the pathogenesis of EHDV-8 infection in red deer. Our results from cases of field suggest that EHDV-8 infection in red deer may have a moderate population-level impact, while being associated with high severity among clinically affected individuals, with sex- and age-related differences that may influence disease detection and clinical outcome. Moreover, although cattle and red deer have been the most affected species in the EHD outbreaks reported in Spain to date, it is imperative to monitor other wild ungulates, including roe deer (*Capreolus capreolus*), fallow deer (*Dama dama*), mouflon (*Ovis orientalis musimon*) and Iberian ibex (*Capra pyrenaica*), as these species also appear susceptible to EHDV infection. Understanding the susceptibility and pathogenicity of native wildlife to emerging orbiviruses is critical for anticipating future outbreaks and for guiding integrated disease management and conservation strategies.

## Supplementary Information

**Additional file 1. Survey for the study of epizootic hemorrhagic diseaseoutbreaks in red deer**. Epidemiological questionnaire (22 questions) used in the online survey conducted between November 2023 and April 2024 to investigate the clinical impact of epizootic haemorrhagic disease virus serotype 8 (EHDV-8) in red deer (*Cervus elaphus*) in Spain.

## Data Availability

The data that support the findings of the present study are available from the corresponding author upon reasonable request.
