# Supplementary material for: Unveiling the clinical signs and pathology in red deer (Cervus elaphus) naturally infected with epizootic haemorrhagic disease virus serotype 8
Source: Vet Res. 2026 Jul 1;57:118. doi: 10.1186/s13567-026-01807-w (PMC13326546; doi:10.1186/s13567-026-01807-w)
Supplement: Supplementary file 1 — Additional file 1. Survey for the study of epizootic hemorrhagic diseaseoutbreaks in red deer. Epidemiological questionnaire (22 questions) used in the online survey conducted between November 2023 and April 2024 to investigate the clinical impact of epizootic haemorrhagic disease virus serotype 8 (EHDV-8) in red deer (Cervus elaphus) in Spain. [file 13567_2026_1807_MOESM1_ESM.pdf]

Survey for the Study of Epizootic Hemorrhagic Disease (EHD) Outbreaks in Red Deer

Epizootic Hemorrhagic Disease (EHD) is a non-contagious infectious viral disease transmitted by vectors (hematophagous dipterans of the genus *Culicoides* spp.), which affects domestic and wild ruminants, especially cattle and red deer (*Cervus elaphus*). This anonymous survey aims to assess the impact of the disease on deer at the national level. It can be completed by managers, gamekeepers, veterinarians, engineers, hunters, landowners, etc., who have encountered suspected cases of EHD. Each survey should refer to a single hunting estate/game reserve/land/property, etc.

1. I give my informed consent to participate in this study and authorize the use of the information collected in this survey for research purposes

   The information collected will be treated confidentially and anonymously and will not be used for any purpose other than that specified in the study description.

   ◯ Yes

   ◯ No

2. Profession

3. Municipality *

4. Province *

5. Total surface area of the hunting estate/property/reserve *

   This response should refer to a single, non-adjacent hunting estate / property / reserve (if more than one is managed).

   The value must be a numeric entry.

6.Does it have perimeter fencing that prevents deer from entering or leaving? *

- ◯ Yes
- ◯ No

7.Estimated total deer density in the hunting estate (number per 100 ha) *

- ◯ 1-10
- ◯ 10-30
- ◯ >30
- ◯ Unknown

8.Percentage of males (including yearlings) in the hunting estate/property/reserve

The value must be a numeric entry.

9.Approximate dates of appearance of the first cases *

10.Approximate dates of peak occurrence of cases *

11.Approximate dates of appearance of the last cases *

12. Have cases reappeared in autumn? *

- ◯ Yes
- ◯ No

13. Do you know the approximate number of deer affected (with clinical signs)? *

- ◯ Yes
- ◯ No

14.Number of affected deer (adult males) *

The value must be a numeric entry.

15.Number of affected deer (adult females) *

The value must be a numeric entry.

16.Number of affected deer (yearlings)*

17.Do you know the approximate number of deer deaths detected? *

- Yes
- No

18.Approximate number of detected deer deaths (adult males) *

The value must be a numeric entry.

19.Approximate number of detected deer deaths (adult females) *

The value must be a numeric entry.

20.Approximate number of detected deer deaths (yearlings) *

The value must be a numeric entry.

21.Observed clinical signs *

☐ Fever

☐ Muzzle erythema (redness)

☐ Udder erythema

☐ Vulvar erythema

☐ Lameness

☐ Muzzle ulcers

☐ Eyelid swelling

☐ Sialorrhea (excessive salivation)

☐ Dyspnoea

☐ Recumbency and apathy

☐ Incoordination

☐ Absence of flight response

☐ Absence of ocular reflex

☐ Alopecia (areas of hair loss)

☐ Abortions

☐ Neonatal death

22.Observations
